# 

**Published:** 1865-01

**Authors:** 


### CHICAGO MEDICAL JOURNAL.

**Terms, $2.00 per Annum.**

### CONTENTS OF THE JANUARY NUMBER.

PAGE.

ORIGINAL COMMUNICATIONS.

Observations on Puerperal Fever. By DeLaskie Miller, M. D., of Chicago ........ 1

The Illinois State Medical Society—Committee on Diseases of the Eye.. 10

Clinical Lectures on Diseases of the Eye. By E. L. Holmes, M. D., of Chicago, Lecturer, on Diseases of the Eye and Ear in Rush Medical College—The Choroid—Anatomy and Physiology........ 11

Typhoid Fever in the 42d Wis. Infantry, stationed at Cairo, Ill. By Geo. D. Winch, Surg. 42d Wis. Infantry........ 15

American Medical Association........ 18

SELECTED.

Scabbies. Read before the Boston Society for Medical Improvement, and Communicated to the *Bost. Med and Surg. Jour.* By James C. White, M. D........ 19

Changes in the Kidneys from Lead Poison........ 32

EDITORIAL AND MISCELLANEOUS.

Compound, Comminuted, Complicated and Impacted Fr..cture of the Right Femur, at Junction of Upper with Middle Third, etc. By A. J. Baxter, M. D., of Chicago........ 33

On the Action of Bromide of Potassium. By S. W. D. Williams M. D., L. R. C. P., London........ 37

Influence of Periosteum on the Reproduction of Bone........ 39

Illustration of some Surgical Phenomena by the Theory of Projectiles. By E. C. Bidwell, Surg. 31st Mass. Vols........ 41

On the Inspiration of Vapor in Certain Lesions of the Breathing Apparatus. By John Hunter, M. A........ 44

